# Progress in the Study of Extraction Methods and Pharmacological Effects of Traditional Chinese Medicine-Derived Carbon Dots

**DOI:** 10.3390/molecules30194015

**Published:** 2025-10-08

**Authors:** Xiaohang Zhou, Junxiang Zhou, Junling Ren, Zhongyuan Qu, Tianlei Zhang

**Affiliations:** 1 Pharmacy College, Heilongjiang University of Chinese Medicine, Heping Road 24, Harbin 150040, China; 2 Pharmacy College, Harbin University of Commerce, Tongda Road 138, Harbin 150040, China; 3 GAP Center, Heilongjiang University of Chinese Medicine, Harbin 150040, China

**Keywords:** TCM carbon dots, preparation method, pharmacological activity

## Abstract

Traditional Chinese medicine-derived carbon dots (TCM-CDs) are prepared by top-down or bottom-up synthesis methods using TCM or their active ingredients as precursors, and the size of TCM-CDs is usually less than 10 nm. It has the advantages of easy preparation, low toxicity, and high compatibility. Compared with traditional Chinese medicines, it shows more outstanding performance in antioxidant, hemostatic, antibacterial, and other aspects, thus having good development prospects. This paper systematically reviews the synthesis methods of carbon dots, focusing on the influence of different traditional Chinese medicine precursors on the formation of carbon dots during the processing process, and analyzes the performance of carbon dots in enhancing the efficacy of original medicinal materials, exerting multi-target synergistic effects, improving bioavailability, and generating new medicinal effects. It is expected to provide a theoretical basis and reference direction for the in-depth research and development of traditional Chinese medicine carbon dots in the field of medicinal value.

## 1. Introduction

Carbon dots (CDs) are zero-dimensional carbon nanomaterials with a size of less than 10 nm, which have a carbon-based skeleton and significant fluorescent properties. The discovery of CDs could be traced back to 2004, when Xu et al. [1] accidentally observed blue-fluorescent carbon particles during the electrophoretic purification of single-walled carbon nanotubes. This discovery was regarded as the prototype of carbon dots. Carbon quantum dots (CQDs) are an important subclass of carbon dots, typically referring to carbon nanoparticles with significant quantum confinement effects and size-dependent luminescent behavior. They were first named by Su et al. [2] in 2006 through laser ablation of graphite and subsequent surface passivation. Compared with traditional semiconductor quantum dots, carbon dots possess excellent biocompatibility, low toxicity, and easy surface functionalization properties, thus demonstrating broad application potential in sensing detection and medical imaging fields [3,4,5,6]. Studies have shown that carbon dots can be applied as drugs or carriers in various biomedical fields such as antibacterial [7,8,9,10,11], anti-cancer [12,13,14,15,16], anti-viral [17,18,19,20], and the treatment of neurological diseases [21,22]. In 2023, the Nobel Prize in Chemistry was awarded to Aleksey Ekimov, Louis Brus, and Moungi Bawendi for their pioneering contributions in the research and synthesis of quantum dots [23]. The awarding of this prize has greatly encouraged the in-depth development of carbon dot-related research, especially in cancer-targeted therapy and intervention for neurodegenerative diseases, opening up new prospects for the development of efficient and precise drugs.

TCM-CDs are carbon dots prepared by top-down or bottom-up methods using Chinese medicinal materials or their active ingredients as carbon sources. Compared with ordinary carbon dots, TCM-CDs not only possess basic advantages such as small size, good water solubility, and ease functional modification [24,25], but also retain the synergistic effect of multi-site and multi-target of traditional Chinese medicine because of their origin from natural Chinese medicine precursors, which significantly improves the intervention effect of complex diseases such as viral infections (e.g., licorice). At the same time, TCM-CDs (such as CDs of Scutellaria baicalensis and Salvia miltiorrhiza) provide the possibility for the development of new drug formulations of traditional Chinese medicine by improving the solubility and bioavailability of poorly soluble components, achieving an important leap from traditional decoctions and powders to modern nano-formulations. Moreover, TCM-CDs usually have higher biocompatibility and lower cytotoxicity, which further enhances their potential for clinical translation [26,27]. Studies have shown that many traditional Chinese medicines, after carbonization and reconfiguration, not only retain their original efficacy but also exhibit enhanced biological activities (like Phellodendron amurense, Panax notoginseng, rhubarb, Perilla frutescens, and Salvia miltiorrhiza), such as hemostasis [28,29,30,31,32,33,34], anti-tumor [35,36,37], anti-inflammatory [38,39,40], antibacterial [41,42,43,44,45], antioxidant [46,47,48,49], and anti-viral [50,51,52] activities, and even new pharmacological functions (such as Aurantii Fructus Immaturus, ginger, and Artemisia argyi). They also have good targeting and high permeability, providing an important approach for the development of efficient and low-toxic new drugs. The following text will systematically elaborate on the relevant content.

## 2. Synthesis of Carbon Dots

In recent years, researchers have constructed diverse carbon dot controllable synthesis systems by selecting precursors, optimizing reaction pathways, and developing novel surface modification structures. Based on the synthesis principle of carbon structures, the preparation techniques are mainly categorized into two types: top-down method and bottom-up method. The two methods have their own characteristics. The former may retain some functional groups of the active ingredients of traditional Chinese medicine, making it suitable for industrial production. However, the particle size uniformity of the former method is poor, the cost is higher, and the experimental conditions are more stringent. The latter method precisely regulates the functional groups and luminescent properties on the surface of carbon dots through precursor molecule design. This method has the advantages of low cost and easy operation. Therefore, the latter is mostly used in the preparation of carbon dots in traditional Chinese medicine.

### 2.1. Top-Down Approach

**Figure 1:**
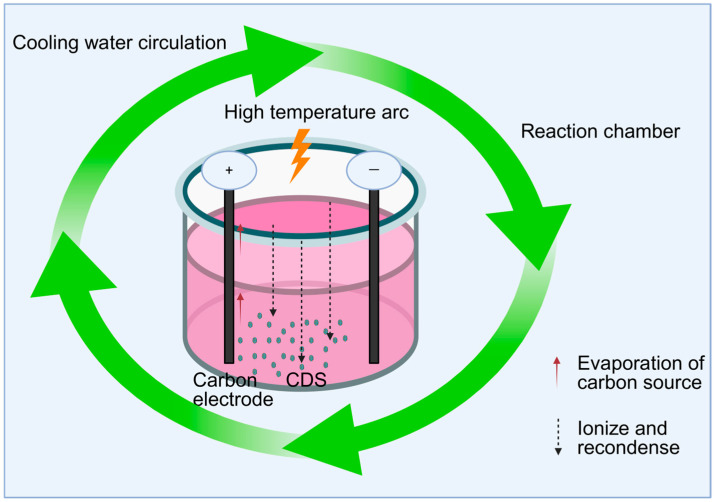
Schematic diagram of the arc discharge method.

The top-down method gradually exfoliates macroscopic carbon materials into nanoscale carbon dots by physical or chemical means. Typical methods include the arc discharge method, electrochemical method, and laser ablation method, etc. (Table 1). The arc discharge method involves applying high-energy electron bombardment between two carbon electrodes to generate arc discharge, which causes the carbon source to evaporate, ionize, and re-condense to form carbon dots. This method is suitable for preparing highly conductive carbon dots (as shown in Figure 1). Bottini et al. [53] isolated carbon dots from raw single-walled carbon nanotubes obtained from different suppliers and those prepared by the nitric acid oxidation arc method, thereby conducting a systematic study on these two types of materials. The electrochemical method applies a voltage or current to the carbon material as an anode in the electrolyte to oxidize and exfoliate it to generate smaller carbon particles. The cost of this method is relatively low compared to other methods. Zhou et al. [54] prepared blue luminescent crystals from multi-walled carbon nanotubes electrochemically for the first time. The laser ablation method generates carbon dots by laser bombardment of carbon targets. This method is suitable for substances that need to retain the active ingredients of traditional Chinese medicine, but it is not suitable for industrial production due to its high cost. Hu et al. [55] synthesized fluorescent carbon nanoparticles by laser irradiation of a carbon powder suspension in organic solvents. Therefore, the top-down method is usually not suitable for preparing traditional Chinese medicine carbon dots. On the one hand, this type of method has a high cost and is difficult to achieve large-scale production; on the other hand, most top-down methods rely on conductive conditions.

### 2.2. Bottom-Up Approach

**Figure 2:**
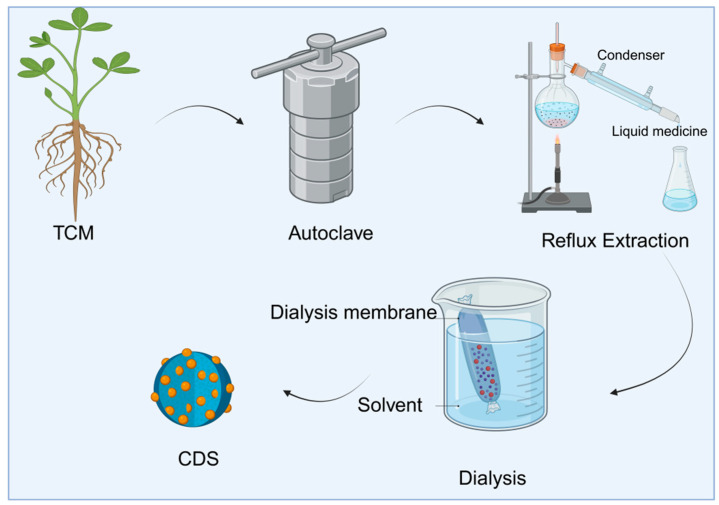
Synthesis path of carbon dots using traditional Chinese medicine as the precursor.

The bottom-up method forms carbon dots with fluorescent properties through reaction paths such as hydrothermal, high-temperature pyrolysis, solvent-thermal, and microwave-assisted carbonization, starting from a small-molecule precursor (Table 2). Similarly, the synthesis of TCM-CDs is primarily based on this method. It uses Chinese medicinal materials as natural precursors and undergoes green treatments, such as hydrothermal or high-temperature pyrolysis, to transform the complex natural chemical components (such as alkaloids, flavonoids, and polysaccharides) in Chinese medicinal materials. These components undergo a series of carbonization and spontaneous surface passivation reactions, including dehydration, polymerization, and aromatization, ultimately forming fluorescent and bioactive carbon dots (as shown in Figure 2).

#### 2.2.1. Hydrothermal Method

The hydrothermal method places traditional Chinese medicines or their extracts in a closed reaction vessel, maintains high temperature and high pressure for a certain period of time, and undergoes chemical reactions such as cracking and polymerization under hydrothermal conditions, gradually forming carbon dots with nanometer-sized particles. For further purification of TCM-CDs, subsequent steps such as centrifugation, filtration, and dialysis are required to remove unreacted raw materials, by-products, and impurities to obtain pure TCM-CDs solution or solid powder. Shen et al. [56] successfully synthesized mint carbon dots with excellent photostability and strong anti-photobleaching properties using mint as the carbon source via a one-step hydrothermal method. The obtained CDs dispersion was transparent yellowish brown and emitted significant blue fluorescence under UV excitation. The study also systematically investigated the influence of hydrothermal conditions on the formation of carbon dots and found that when the reaction temperature was lower than 120 °C or the time was shorter than 8 h, uncarbonized polymer structures could be observed under transmission electron microscopy, indicating that the carbon source was not completely converted; while after reacting at 180 °C for 8 h, microscopic characterization showed no polymer residues in the product, and the carbon source had been completely pyrolyzed. The results indicated that reaction temperature and time are key factors for successful preparation. Zhao Huali et al. [57] also optimized the reaction temperature and time to achieve the ideal carbonization effect of Radix Vladimiriae. It was shown that if the reaction time was not long enough, the carbonization process could not be carried out adequately, while if the reaction time was too long, the fluorescent carbon dots were prone to agglomeration. Dong et al. [58]. synthesized carbon dots with high quantum yield and controllable wavelength photoluminescence by hydrothermal method and demonstrated that the photoluminescent properties of the obtained dots could be regulated by controlling the reaction temperature. In addition, Wang et al. [59]. prepared carbon dots with excellent fluorescence quantum yield by a one-step hydrothermal method using papaya as a carbon source, which can be directly used as a probe for iron sensing and detection without modification, providing a green pathway for the preparation of TCM-CDs.

#### 2.2.2. High-Temperature Pyrolysis

High-temperature pyrolysis is a method in which the carbon source is heated at high temperature for a long period of time in an inert gas or in an atmospheric environment, so that it undergoes thermal decomposition, carbonization, and condensation to form carbon dots. Dager et al. [60]. prepared fennel fruit carbon dots that emit blue fluorescence under ultraviolet irradiation (365 nm) by pulverizing fennel seeds and then placing them in crucible cups and continuously heating them with a heating plate at 500 °C. Wang et al. [61] synthesized Pueraria lobata radix carbon dots by pyrolysis at 300 °C and used them to study their anti-gout effect. This method does not require solvents and is generally suitable for herbs that are not sensitive to heat and can be produced on a large scale, but the high temperature evaporates the water, and the product is more hydrophobic and requires subsequent treatment to improve solubility.

#### 2.2.3. Microwave-Assisted Method

Microwave-assisted carbonization utilizes microwave radiation to heat the precursor rapidly and uniformly and triggers the carbonization reaction through heat generated by intermolecular friction. Tsai et al. [62]. obtained CDs by placing gardenia fruits and gardenia flower charcoal in a bottle, adding a solvent, performing electromagnetic stirring and heating, transferring them to a conical flask, then treating them with a 70 W microwave for 5 min, cooling, filtering, and freeze-drying the solution. Zhu et al. [63] dissolved polyethylene glycol and sugars in different ratios in distilled water to obtain a transparent solution. After heating in a microwave oven for 2–10 min, the color of the solution gradually changed from colorless to yellow and dark brown, indicating the generation of CDs, which proved that the microwave-assisted method can rapidly synthesize CDs within a few minutes. This method can precisely control the reaction time, has short time consumption, and improves preparation efficiency.

#### 2.2.4. Solvothermal Method

The solvothermal method uses organic solvent as reaction media to dissolve, carbonize, or crystallize carbon sources under high temperature and airtight, high-pressure conditions. Its core advantage lies in the multiple selectivities of solvents. By choosing solvents of different polarities and boiling points, such as ethanol and toluene, the rate of the reaction system and surface chemical properties of the products can be precisely regulated. Fan Jinhao et al. [64]. prepared Rhodiola rosea water-soluble carbon dots (RWW-CDs) and Rhodiola rosea alcohol-soluble carbon dots (RWE-CDs) by this method, and found that the size of the RWE-CDs was larger, while the fluorescence stability of the RWW-CDs was better. Jiaxin Li [65] et al. used lotus leaves as the carbon source and added organic solvent (formamide) to produce carbon dots with a particle size of 2.45 nm at high temperature in a muffle furnace, which were used to detect the content of sunset yellow in beverages. Compared with the hydrothermal method, most of the solvents used in this method are toxic. Therefore, for the synthesis of carbon dots using traditional Chinese medicine as precursors, the hydrothermal method is mostly adopted.

#### 2.2.5. Other Methods

In addition to the above four methods, there is also the template method, which guides the size and morphology of carbon dots by using porous materials (such as mesoporous silica) or molecular templates. Liu et al. [66]. used soluble phenol–formaldehyde resin as the carbon source, prepared satellite polymer/F127/silica composites through the aqueous-phase method. The silica carrier was etched with NaOH solution, and then acid treatment and surface modification were carried out successively. Finally, polychromatic photoluminescent CDs with good water dispersibility were obtained. Liao [67] et al. successfully prepared a series of bismuth oxychloride composite photocatalysts containing carbon dots by adding Panax ginseng powder as a templating agent in a solvent-thermal synthesis system of bismuth oxychloride, thus enhancing the photocatalytic performance. The size and structure of CDs synthesized by this method can be precisely controlled. In addition, there is also the acid dehydration method, which refers to the method of forming CDs by using carbon-containing organic materials as precursors, mixing them with concentrated acid (concentrated sulfuric acid, phosphoric acid, etc.) and then heating, so that the carbon source undergoes a condensation and carbonization reaction through the dehydration and catalytic effect of the acid. Peng [68] et al. used carbohydrates as raw materials to prepare carbon-containing materials by dehydration with concentrated sulfuric acid, then decomposed them into nanoparticles by nitric acid treatment, and finally passivated to obtain luminescent carbon dots. This method is fast and mild, and the performance of carbon dots can be regulated by adjusting the type and concentration of acid, but the corrosiveness of strong acid and the disposal of waste acid need attention.

It is worth noting that Chinese medicines are usually processed before the synthesis of CDs. This traditional processing technology directly affects the reactivity of precursors and the properties of products by altering the texture of medicinal materials and the composition and proportion of chemical components (e.g., reducing toxicity, enhancing active ingredients, or generating new compounds). Compared with single-molecule precursors such as citric acid [69] and phenylenediamine, carbon dots synthesized using charcoal drug as precursors may have more regulated doping types, surface functional group distributions, and biological activities. They can achieve self-doping (N, S, O) and multi-functional group surface modification without complex post-modification. This not only retains the enhanced or new pharmacological activity of processing but also shows significant advantages in biocompatibility and multi-target therapy, which provides an important basis for the subsequent study of the effect of processing on the formation mechanism of carbon dots and the regulation of pharmacological activity.

## 3. Effects of Processing on Carbon Dots and Their Pharmacological Research

### 3.1. Enhanced Original Efficacy

#### 3.1.1. Phellodendri Cortex (Huangbo)

Huangbo, recorded in *Shennong’s Classic of Materia Medica* [70], is the dried bark of *Phellodendron chinense* Schneid., a plant of the Rutaceae family. It is mainly produced in Sichuan, Guizhou, and other places, and is generally known as “Sichuan yellow cypress”. It is bitter and cold in nature and belongs to the kidney and bladder meridians. Its effects include clearing heat and drying dampness, removing fire and vapor, detoxifying and healing sores, and is used in treating damp-heat diarrhea, jaundice and urinary redness, tidal fever, night sweating, eczema, and wet sores [71].

##### The Influence of the Preparation Process on the Carbon Dots of Phellodendri Cortex

The commonly used processed products of Phellodendri Cortex include wine-processed Phellodendri Cortex and charred Phellodendri Cortex. After undergoing charring, the medicinal effects of this drug change from primarily focusing on clearing heat and purging fire to astringing to stop bleeding and enhancing the effect of astringing the intestines to relieve diarrhea. Modern research has found that when Phellodendri Cortex is charcoal-fried, alkaloids are demethylated to form quinoline polycyclic aromatic hydrocarbons (e.g., berberrubine), and lignin is carbonized to form graphite microcrystals. Li et al. [72] analyzed the dynamic changes in the chemical components of Phellodendri Cortex before and after charring based on mass spectrometry and found that chemical components such as berberine and chlorogenic acid decreased significantly, while the contents of components such as tetrahydrocoptisine and berberrubine increased significantly. Similarly, Xu Yali et al. [73] compared the chemical components of salt-processed Phellodendri Cortex and charred Phellodendri Cortex, confirming that the content of berberine hydrochloride in charred Phellodendri Cortex was relatively low, while the content of berberrubine was significantly increased. This indicates the transformation of the material basis for the medicinal effects of Phellodendri Cortex after charring. Dai Qi et al. [74] found that, compared with wine-processed Phellodendri Cortex and salt-processed Phellodendri Cortex, charred Phellodendri Cortex lacks antioxidant activity but has enhanced hemostatic effect.

##### Pharmacological Activity of Phellodendri Cortex Carbon Dots

Modern scientific studies have shown that carbon dots extracted from the traditional Chinese medicine Phellodendri Cortex have significant pharmacological activities, especially in the hemostatic mechanism. Liu et al. [75]. found that the fibrinogen (FIB) and platelet (PLT) contents of SD rats treated with a solution of PCC-CDs (Phellodendri Cortex Carbonisatus-carbon dots) were significantly increased, and the content of the prothrombin time (TT) decreased. An increase in FIB and a decrease in TT could promote the conversion of fibrinogen to fibrin, accelerating blood coagulation; increased PLT content help enhance thrombus formation and plug broken blood vessels. These combined effects indicate that PCC-CDs synergistically promote blood coagulation and thus hemostasis through multiple pathways, as shown in Table 3.

**Figure 3:**
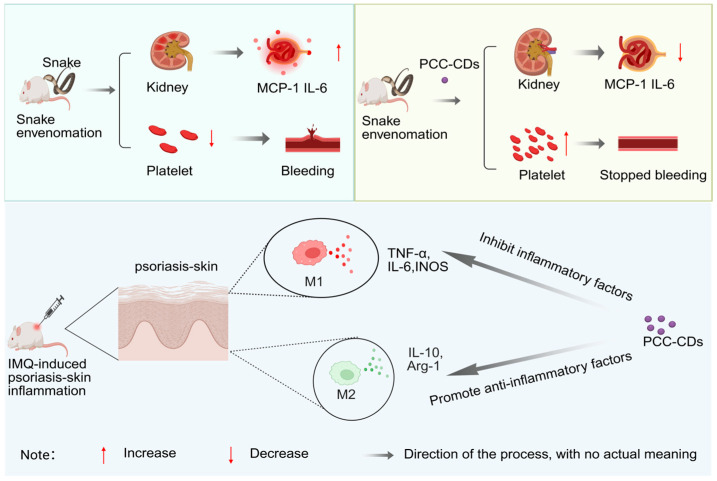
Mechanism of action of PCC-CDs in patients with acute kidney injury and psoriasis induced by the five-step snake venom.

Similarly, Zhang et al. [76]. synthesized PCC-CDs by one-step pyrolysis of Phellodendri Cortex as a carbon source at a high temperature of 350 °C. Studies have shown that after envenomation by Deinagkistrodon acutus (the five-step viper), symptoms such as hemorrhage and thrombocytopenia occur. Additionally, renal lesions develop, and levels of inflammatory factors including IL-10, MCP-1, and IL-1β become abnormal. After administration of PCC-CDs, the levels of indicators such as serum creatinine (SCR), blood urea nitrogen (BUN), urinary total protein (UTP), and microalbuminuria (MALB) are alleviated. Meanwhile, the levels of the chemokine MCP-1 and the pro-inflammatory cytokine IL-1β decrease. This indicates that PCC-CDs can regulate acute kidney injury (AKI) induced by Deinagkistrodon acutus venom through pathways such as inhibiting the inflammatory response, improving renal function, and increasing platelet count. Furthermore, studies have shown that patients with psoriasis exhibit phenomena such as epidermal hyperplasia, parakeratosis, erythema, scaling, and skin thickening, along with abnormal levels of inflammatory factors including IL-10, TNF-α, and IL-6. After topical application of PCC-CDs, skin symptoms (such as erythema, scaling, and infiltration) and the Psoriasis Area and Severity Index (PASI) score were alleviated. Meanwhile, levels of M1-type inflammatory mediators (TNF-α, IL-6, iNOS) in skin tissues were reduced, and the levels of M2-type anti-inflammatory mediators (IL-10, Arg-1) increased. This indicates that PCC-CDs can inhibit the inflammatory response by regulating the macrophage M1/M2 polarization pathway [77] (Figure 3).

In summary, modern pharmacology has shown that the bioactive components (including alkaloids and flavonoids) in Phellodendri Cortex undergo significant changes after being treated with traditional processing techniques such as high-temperature carbonization and stir-frying, which make its original antioxidant activity to nearly disappear, showing new medicinal value. On the one hand, PCC-CDS can activate the FIB system, accelerate platelet aggregation, and exert hemostatic effects. On the other hand, they significantly repair kidney tissue and skin damage caused by inflammation.

#### 3.1.2. Panax Notoginseng (Sanqi)

Sanqi is the root and rhizome of *Panax notoginseng* (Burk.) F. H. Chen in the Araliaceae family. It is warm in nature, sweet and slightly bitter in taste, and acts on the liver and stomach meridians in TCM theory. It exerts pharmacological effects such as hemostasis, anti-inflammation, and promotion of tissue repair [71]. Panax notoginseng has the elegant name “Jin Buhuan” (literally meaning “invaluable” or “not to be exchanged for gold”), and its origin can be traced back to the “*Compendium of Materia Medica*” by Li Shizhen [78]. Meanwhile, the output value of Panax notoginseng-related products exceeds CNY 70 billion; there are 513 types of Chinese patent medicine preparations using notoginseng as a raw material and more than 3600 pharmaceutical approval documents for such medicines. Thus, Panax notoginseng enjoys broad market applications [79].

##### The Impact of the Preparation on the Carbon Dots of Panax Notoginseng

The earliest record of the processing of Sanqi can be found in the *Wan Shi Nu Ke* of the Ming dynasty [80]. After processing, the medicinal property of this herb changes and tends to be warm in nature. Although it still retains a certain degree of hemostatic and blood stasis-resolving ability, its effect on blood nourishment is more prominent. Currently, the common processing methods available on the market are roughly divided into two types: oil-frying (frying with oil until the surface turns slightly yellow) and steaming (steam thoroughly over water, then slicing and drying) [81]. Yu Heshui et al. [82]. analyzed the chemical composition of processed Sanqi products and found that after processing, triterpene saponins mainly undergone glycosylation hydrolysis and dehydration reactions. Du [83] compared the volatile components in raw and processed Sanqi by using headspace gas chromatography–mass spectrometry (HS-GC-MS). The study confirmed that the relative content of pinene in processed Panax notoginseng was lower, which led to the weakening of its anti-inflammatory pharmacological activity. Meanwhile, a variety of Maillard intermediate reaction products were newly generated; most of these products were nitrogen-containing compounds, which provided abundant nitrogen-doped carbon precursors for carbon dot synthesis. Zheng [84] et al. successfully prepared two types of derived carbon dots (i.e., Panax notoginseng-based carbon dots and Panax notoginseng-based highly fluorescent nitrogen-doped carbon dots) via a simple hydrothermal method, which were used for rapid detection of Cr^6+^.

##### Pharmacological Activity of Panax Notoginseng Carbon Dots

**Figure 4:**
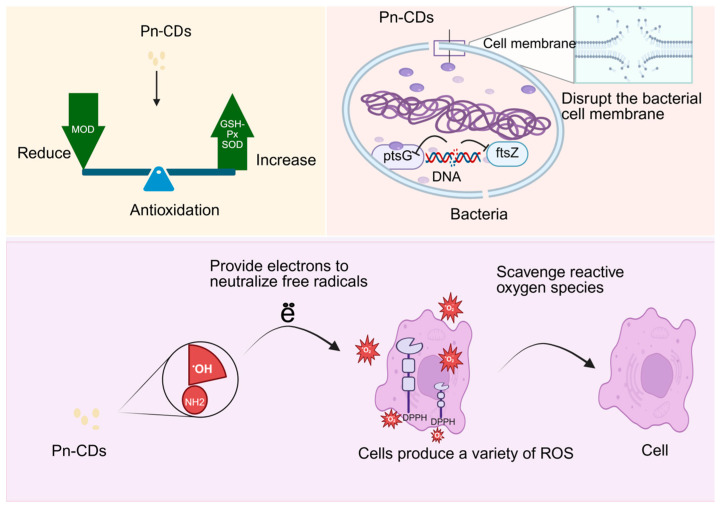
Mechanism of bacteriostatic and antioxidant effects of Pn-CDs.

Carbon dots extracted from Panax notoginseng exhibit great potential for bacteriostasis and antioxidant activities due to their unique optical properties, good biocompatibility, and low toxicity. Xiaodan Zheng [85] successfully prepared Panax notoginseng-based carbon dots (Pn-CDs) and N-doped Panax notoginseng-based carbon dots (Pn N-CDs) via a one-step hydrothermal method. The study revealed that Pn-CDs exhibit significant antibacterial and antioxidant activities. In terms of antibacterial effects, Pn-CDs achieve antibacterial activity primarily by disrupting the integrity of bacterial cell membranes, inhibiting DNA biosynthesis, and interfering with the expression of glucose transport-related gene (ptsG) and cell division gene (ftsZ), thereby blocking energy supply and bacterial proliferation. On the one hand, its antioxidant activity can directly provide electrons to neutralize free radicals (e.g., DPPH and superoxide anions) through surface hydroxyl and amino functional groups to scavenge reactive oxygen species. On the other hand, it can significantly upregulate the activities of superoxide dismutase (SOD) and glutathione peroxidase (GSH-Px) to repair oxidative damage to tissues, while inhibiting lipid peroxidation and reducing malondialdehyde (MDA) content to alleviate oxidative damage to cell membranes. Together, these effects achieve the goal of inhibiting bacteria by reducing oxidative damage to the cell membrane and exerting antioxidant activity, as shown in Figure 4.

#### 3.1.3. Rhubarb (Dahuang)

Dahuang refers to the dried roots and rhizomes of *Rheum palmatum* L., *Rheum tanguticum* Maxim. ex Balf., or *Rheum officinale* Baill. of the Polygonaceae family [71]. In TCM theory, rhubarb is bitter in taste and cold in nature, and it exerts effects such as purging accumulation (by promoting bowel movements), clearing heat, purging fire, cooling blood, and detoxifying.

##### The Influence of the Preparation on the Carbon Dots of Rhubarb

Rhubarb, as a Chinese herb commonly used in clinical practice for the treatment of solid-heat conditions, is mostly used to relieve constipation. After rhubarb is carbonized, its effects of clearing heat and cooling blood, as well as its purging effect, are transformed into hemostatic and blood stasis-resolving effects. Studies have shown that the chemical components of rhubarb undergo transformation during its processing. Cai Xinjie [86] determined the chemical composition of rhubarb charcoal by the HPLC method and found that a new substance, 5-hydroxymethylfurfural, was generated. At the same time, Wei Jiangcun et al. [87]. found that the total flavonoid and total anthraquinone contents of rhubarb charcoal significantly decreased (Table 4). Guo Dongyan [88] further investigated the changes in chemical composition of rhubarb before and after frying and found that the contents of rhubarb phenol, rhubarbol, rhubarbic acid, and rhubarbin methyl ether decreased after frying rhubarb charcoal (Table 4). These components are the main active ingredients responsible for the purgative and accumulation-resolving effects. A decrease in their contents indicates that this effect weakens and the pharmacodynamic material basis undergoes a transformation. Li Li [89] conducted an in-depth study on the variation in the material basis of Dahuang before and after processing. The results showed that charred rhubarb has almost no purgative or antipyretic effects; instead, it exhibits an enhanced hemostatic effect. In traditional medicine, it is commonly used to treat bleeding symptoms. Therefore, charred rhubarb can be used as a precursor for carbon dots with hemostatic properties.

##### The Pharmacological Activity of Rhubarb Carbon Dots

**Figure 5:**
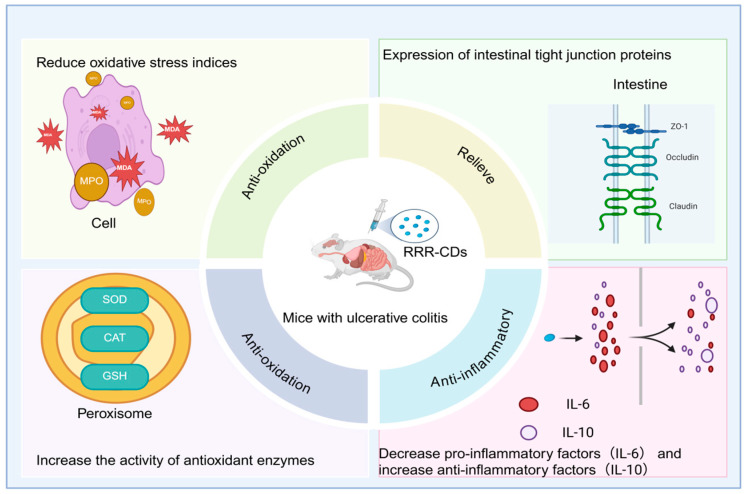
Diagram of the mechanism of RRR-CDs against ulcerative colitis.

Modern research has shown that rhubarb carbon dots (RRR-CDs) extracted from charred rhubarb exhibit significant potential in the treatment of ulcerative colitis (UC). Zhang et al. [90] established a mouse UC model through dextran sulfate sodium (DSS) induction. The mice showed typical symptoms such as weight loss, diarrhea, and hematochezia, along with destruction of the colonic mucosal structure, loss of crypts, and infiltration of a large number of inflammatory cells. At the molecular level, in the model group, the levels of pro-inflammatory factors (TNF-α, IL-6) in mice increased significantly, the level of the anti-inflammatory factor (IL-10) decreased, the indicators of oxidative stress (MPO, MDA) rose, the activities of antioxidant enzymes (SOD, CAT, GSH) declined, and the expression of intestinal tight junction proteins (ZO-1, Occludin, Claudin-1) was significantly reduced. After intervention with RRR-CDs, the trend of weight loss in mice was improved, the disease activity index (DAI) score decreased significantly from 2.8 to 1.6, colon shortening was inhibited, histopathological damage was significantly alleviated, and the above-mentioned indicators related to inflammation, oxidative stress, and intestinal mucosal barrier function were all effectively reversed. These results indicate that RRR-CDs significantly enhance the therapeutic effect on ulcerative colitis by synergistically exerting multiple pharmacological mechanisms such as hemostasis, antioxidation, anti-inflammation, and protection of the intestinal mucosal barrier. Compared with raw rhubarb, RRR-CDs show better anti-inflammatory activity and overall therapeutic effect, highlighting the potential application value of traditional Chinese medicine carbon dots in enhancing anti-inflammatory effects, as shown in Figure 5.

#### 3.1.4. Schizonepetae Herba (Jingjie)

Jingjie refers to the dried aerial parts of *Schizonepeta tenuifolia* Briq., a plant belonging to the Lamiaceae family [71]. In terms of efficacy, it mainly exhibits two prominent effects: dispelling wind and relieving exterior syndrome, and promoting rash eruption and relieving itching. Jingjie has a long history of use as a medicinal herb, which is recorded in numerous traditional Chinese medicine classics, and it is one of the commonly used herbs in the clinical practice of traditional Chinese medicine

##### The Influence of Processing on Schizonepetae Herba Carbon Dots

Schizonepetae Herba is commonly used as an exterior-releasing herb in clinical practice, mainly for treating conditions such as common cold, headache, measles, and early-stage rubella. After undergoing high-temperature carbonization, the efficacy of Schizonepetae Herba transforms into an astringent and hemostatic effect. Schizonepetae Herba and charred Schizonepetae Herba exhibit significant differences in efficacy, indicating that the types and contents of their chemical components have undergone changes during the charring process. Wang Gexiang [91] conducted a quantitative analysis of the active components of Schizonepetae Herba before and after processing using fingerprint chromatography. The results showed that after being processed into charred form, the contents of pulegone, rosmarinic acid, and menthone decreased, while the content of caffeic acid increased. Similarly, Chu Zhaozheng [92] also studied the component changes in Schizonepetae Herba after charring and confirmed that during the charring process, original components such as hesperidin, rosmarinic acid, and pulegone gradually degraded and new substances were produced. Modern pharmacological studies have shown that Schizonepetae Herba contains components such as volatile oils (e.g., pulegone) and flavonoids, which exhibit diaphoretic, anti-inflammatory, anti-allergic, and bacteriostatic effects. After processing, the contents of these components gradually decrease; at the same time, new substances are generated, which possess a certain hemostatic effect—thus confirming the transformation of its pharmacological effects [93]. These transformations provide an ideal precursor for the preparation of carbon dots.

##### Pharmacological Activity of Schizonepetae Herba Carbon Dots

Currently, studies on the hemostatic mechanism of charred TCM drugs mainly focus on the coagulation and anticoagulation systems, fibrinolytic system, and platelet reaction [94]. It has been found that there are certain similarities in composition and structure between Schizonepetae Herba carbon dots and charred Schizonepetae Herba, and thus both of them can exert hemostatic effects. Zhang et al. [95]. extracted Schizonepetae Herba carbon dots (SHC-CDs) from charred Schizonepetae Herba. In a mouse tail amputation and liver scratch model, administration of SHC-CDs resulted in decreased prothrombin time (PT) and increased FIB content, whereas activated partial thromboplastin time (APTT) and TT were not significantly different from those of the control group, suggesting that SHC-CDs inhibit hemorrhage via promotion of exogenous coagulation pathways and activation of the FIB system. Similarly, Sun [96] et al. prepared Schizonepetae Herba carbon dots by a modified pyrolysis method. In a mouse tail amputation and liver scratch model, the bleeding time was significantly shortened and platelet counts were significantly increased after administration of SHC-CDs, indicating that SHC-CDs could inhibit hemorrhage by elevating platelet counts. Subsequently, in the Deinagkistrodon acutus venom model, SHC-CDs were administered. It was found that the platelet count increased significantly at multiple time points, while the hemorrhagic activity decreased significantly. This proves that SHC-CDs can regulate the excessive platelet consumption induced by snake venom by increasing the number of platelets, thereby inhibiting the hemorrhagic activity of the snake venom and alleviating bleeding symptoms, as shown in Table 5.

It can be seen that SHC-CDs can promote exogenous coagulation, activate the FIB system, convert fibrinogen into fibrin, and promote blood coagulation. At the same time, SHC-CDs can also elevate platelet numbers and enhance the role of platelets in the coagulation process. Through the synergistic action of these modalities, the effect of hemostasis is finally achieved.

#### 3.1.5. Salvia Miltiorrhiza (Danshen)

Salvia miltiorrhiza refers to the dried roots and rhizomes of *Salvia miltiorrhiza* Bge., a plant belonging to the Lamiaceae family [71]. It is mainly produced in Sichuan, Shandong, Henan, and other regions in China, among which “Chuan Danshen” is recognized as an authentic medicinal material. In terms of traditional Chinese medicine properties, it is bitter in taste and slightly cold in nature, and has a meridian tropism for the heart and liver meridians. It is distinguished by its effects of promoting blood circulation to remove blood stasis, dredging meridians to relieve pain, and clearing the heart to resolve restlessness. It is widely used for treating conditions such as chest impediment (a TCM term roughly corresponding to angina pectoris in modern medicine), irregular menstruation, dysmenorrhea and amenorrhea, and abdominal masses (e.g., cirrhosis), as well as palpitation and insomnia. There has long been a reputation in TCM that “One herb of Danshen is equivalent in efficacy to the Four Substances Decoction (Siwu Decoction)”

##### The Influence of Processing on Salvia Miltiorrhiza Carbon Dots

Studies have shown that the chemical composition of Salvia miltiorrhiza is transformed after processing. Lu Xiaohua [97] compared the contents of salvianolic acid B in different processed products of Salvia miltiorrhiza and found that the content was the lowest in charred Salvia miltiorrhiza. Similarly, Cheng Lifang [98] compared the content of tanshinone IIA in different processed products, and also showed that the content was the lowest in charred Salvia miltiorrhiza (Table 6). Modern pharmacological studies have shown that Salvia miltiorrhiza contains active components such as tanshinones and salvianolic acids, which exhibit effects including improving microcirculation, anti-thrombotic, anti-oxidative, anti-fibrotic, and protecting the cardiovascular and cerebrovascular systems. After high-temperature carbonization, although the content of some active components decreases, its core pharmacological effects are still retained. Wang Peiqing [99] found that the ethyl acetate fraction of charred Salvia miltiorrhiza showed the most prominent free radical scavenging capacity. Furthermore, after Salvia miltiorrhiza is processed into charred form, the porous properties in its carbonized structure make it an ideal precursor for the preparation of functionalized carbon dots.

##### Pharmacological Activity of Salvia Miltiorrhiza Carbon Dots

**Figure 6:**
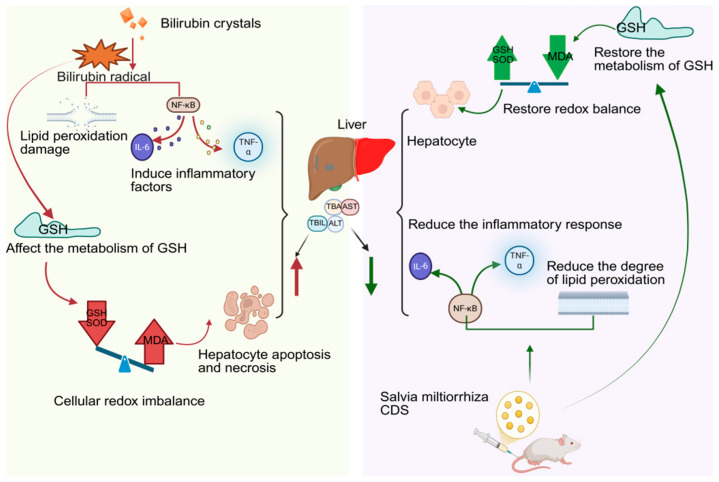
Mechanism of action of Salvia miltiorrhiza carbon dots in the treatment of liver injury due to hyperbilirubinemia.

CDs derived from Salvia miltiorrhiza not only retain the active molecules but also exhibit significantly enhanced antioxidant capacity compared to the raw medicinal material (Table 6). Li et al. [100] successfully prepared Salvia miltiorrhiza CDs using an improved pyrolysis method. Studies have shown that elevated levels of reactive oxygen species (ROS) in plants disrupt redox balance, triggering lipid peroxidation, protein denaturation, and DNA damage, thereby inhibiting plant growth. Salvia miltiorrhiza carbon dots, with their surface rich in functional groups such as phenolic hydroxyl groups, can directly scavenge DPPH, superoxide anions, and hydroxyl radicals, and mimic the activity of enzymes like SOD to catalyze the conversion of harmful ROS. Their nanoscale size also enables them to easily penetrate plant cells, reduce membrane lipid peroxidation, and maintain ion balance, thereby effectively alleviating oxidative damage caused by salt stress, increasing biomass, and reducing MDA content in plants. In addition, Guo et al. [101] found that carbon dots extracted from Salvia miltiorrhiza charcoal showed potential in protecting against liver damage induced by hyperbilirubinemia. Hyperbilirubinemia can lead to abnormally elevated bilirubin levels in the body; excessive bilirubin promotes free radical generation, induces lipid peroxidation, upregulates the expression of inflammatory factors (such as IL-6 and TNF-α), disrupts glutathione (GSH) metabolism, causes oxidative stress imbalance, and leads to hepatocyte apoptosis and necrosis, which is manifested by increased serum levels of ALT, AST, TBA, and TBIL. After intervention with Salvia miltiorrhiza carbon dots, the above serum indicators and levels of inflammatory factors were alleviated, the MDA content in liver tissue decreased, and the activities of SOD and GSH increased. These results indicate that Salvia miltiorrhiza CDs can improve hyperbilirubinemia-related liver damage by inhibiting inflammatory responses, reducing the release of inflammatory factors, enhancing antioxidant enzyme activity, and alleviating lipid peroxidation (Figure 6).

### 3.2. Synergistic Effects at Multiple Sites and Multiple Targets

#### 3.2.1. Licorice (Gancao)

Licorice (Gancao) is the dried root and rhizome of *Glycyrrhiza uralensis* Fisch., *Glycyrrhiza inflata* Bat., or *Glycyrrhiza glabra* L. of the legume family [71]. The drug was first published in *Shennong’s Classic of Materia Medica* and has the effect of prolonging life [102]. According to records in *Compendium of Materia Medica*, this herb (referring to licorice) can harmonize the effects of other herbs and neutralize toxins from plants, minerals, and metals, thus being known as the “monarch” among hundreds of herbs [103]. Furthermore, among the 1493 kinds of Chinese patent medicines recorded in *Pharmacopeia of the People’s Republic of China* (ChP), more than 60% contain licorice as an ingredient.

##### The Influence of the Processing on the Carbon Dots of Licorice

Studies have shown that licorice exhibits effects such as detoxification, regulating the middle energizer, relieving cough and asthma, and alleviating pain and spasm. After high-temperature carbonization, it is converted into charred licorice. This process not only changes its physical form but also transforms its original effects—such as relieving cough and asthma and harmonizing the effects of other herbs—into focusing mainly on hemostasis. Li Benchun et al. [104]. found that the content of glycyrrhizin and glycyrrhizic acid decreased significantly after charring. Modern pharmacological studies have shown that the active components (e.g., flavonoids) in licorice are partially decomposed at high temperatures to generate carbonaceous structures with higher adsorption capacity, thus exerting hemostatic efficacy. The transformation of these components and efficacy also lays the foundation for further research and application of licorice charcoal. Based on this, Zhang et al. [105] charred licorice and extracted licorice carbon dots from it. These licorice carbon dots can serve as a potential clinical drug for the treatment of menopausal syndrome. This finding reveals that licorice has developed new therapeutic effects after undergoing charring processing.

##### The Pharmacological Activity of Licorice Carbon Dots

**Figure 7:**
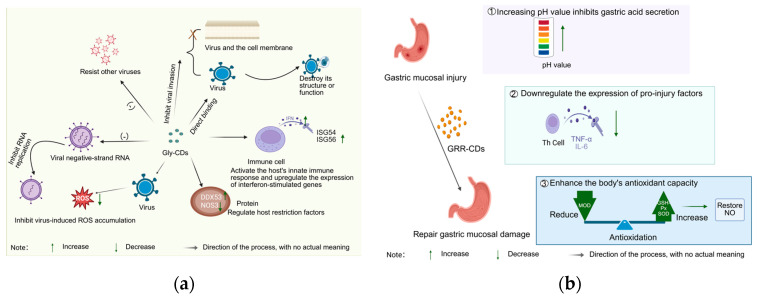
(**a**) Mechanism of anti-ulcer action of licorice carbon dots; (**b**) Mechanism of synergistic anti-viral action at multiple sites of Gly-CDs.

Licorice, as the core component of “harmonizing all medicines”, has recently been shown to be used as a green precursor molecule for the synthesis of TCM-CDs. Licorice carbon dots are often used as the drug of choice in treating ulcers. Liu et al. [106]. extracted a licorice carbon solution (GRRC-NCs) from charred licorice. Using stress-induced gastric ulcers in rats as the animal model, after intra-gastric administration of GRRC-NCs, they confirmed that GRRC-NCs inhibit gastric acid secretion by increasing the pH value. Meanwhile, it downregulates the expression of pro-damaging factors (i.e., reducing the levels of TNF-α and IL-6) and regulates cellular stress response pathways, thereby alleviating the degree of gastric mucosal damage and significantly improving ulcers. Liu et al. [107] directly used licorice as a carbon source, and its carbon dots (Gly-CDs) were synthesized and characterized using one-step pyrolysis. The carbon dots had a spherical structure with a large number of active groups on the surface, and the particle size was in the range of 2–10 nm. The study showed that, under the effect of alcohol and other factors, mice showed gastric mucous membrane damage, and the gastric tissues of mice showed lesions, and the indexes such as MDA, SOD, and nitric oxide (NO) in the serum and the gastric tissues showed abnormalities [108]. After the administration of Gly-CDs, the MDA content in serum and gastric tissues was reduced, the SOD activity was increased, the NO content in gastric tissues was normalized, the gastric mucosal damage was reduced, and the ulcer index was reduced, indicating that Gly-CDs can improve the antioxidant ability of the body and regulate the level of NO in the gastric tissues to restore the gastric mucosal damage induced by free radicals in the metabolism process of alcohol and thus play an anti-ulcer effect (Figure 7a).

In the anti-viral field, licorice and Gly-CDs exhibit different action characteristics. Licorice mainly relies on extracts and active sites to intervene in the viral replication cycle and modulate the inflammatory response, immune function, and oxidative stress state of the body. Li Zhongyuan et al. [109]. screened the active sites against respiratory syncytial virus (RSV) as n-butanol and aqueous sites by investigating the inhibitory effects of licorice on RSV, HSV-1, EV71, and other viruses. These active sites act on the adsorption, penetration, and replication phases of the RSV replication cycle, and can effectively improve lung tissue damage. Their mechanisms of action include: inhibiting the TLR4/NF-κB pathway to reduce inflammatory responses; increasing IFN-β levels to inhibit RSV immune escape; and reducing the level of oxidative stress with the help of the Keap1/Nrf2 pathway to protect lung tissue. In contrast, Gly-CDs focus on multi-site inhibition of viruses (Table 7). Tong et al. [110] synthesized Gly-CDs by hydrothermal method using the active ingredient of licorice (glycyrrhetinic acid) as a precursor molecule. It has been demonstrated that Gly-CDs can directly bind to viral particles, destroying the structure or function of the virus, and inactivating the virus. In the process of virus invasion into host cells, Gly-CDs can block the binding of viruses to receptors or interfere with the fusion of virus and cell membrane, thus inhibiting virus invasion. Inside the cell, Gly-CDs effectively block viral replication by reducing the synthesis of viral negative-strand RNA and inhibiting the expression of key viral proteins (e.g., the N protein and nsp2 of porcine reproductive and respiratory syndrome virus (PRRSV). In addition, Gly-CDs can activate the innate immune response of the host, upregulate the expression of interferon-stimulated genes (e.g., ISG-54, ISG-56, etc.), and enhance the anti-viral ability of the organism. At the same time, it inhibits virus-induced ROS accumulation, maintains intracellular redox balance, and reduces cellular damage caused by oxidative stress. In terms of regulating host-restriction factors, Gly-CDs can upregulate the expression of the anti-viral protein DDX53 and downregulate the expression of the pro-viral protein NOS3, further restricting viral infection and transmission. It is worth mentioning that the anti-viral effect of Gly-CDs is broad-spectrum, and it is effective against a variety of viruses such as PRRSV, porcine pseudorabies virus PRV (DNA virus), and porcine epidemic diarrhea virus PEDV (RNA virus), demonstrating a unique advantage of synergistic anti-viral action in multi-targets. This is mainly due to the fact that Gly-CDs have a large surface area and abundant contact sites, which can have multivalent interactions with viruses and inhibit viruses from various aspects, as shown in Figure 7b.

The study of pharmacological effects of licorice carbon dots is a new direction in the modernization research of traditional Chinese medicine. Although traditional licorice extracts have anti-inflammatory, antioxidant, and immunomodulatory effects, they have limitations such as low bioavailability and poor targeting. However, licorice carbon dots can not only achieve the pharmacological effects of high anti-viral activity by inhibiting the multi-site mechanism and regulation of body functions, but also clearly elucidated the mechanism of action of licorice as a preferred drug for anti-gastric ulcers, which provided a new way of thinking in the study of the effective material basis of licorice.

### 3.3. Improve Bioavailability

#### 3.3.1. Moutan Cortex (Mudanpi)

Mudanpi, also known as peony bark, is the dried root bark of *Peony Paeonia suffruticosa* Andr. of the buttercup family [71]. Its nature and flavor are bitter, pungent, and slightly cold, and it enters the heart, liver, and kidney meridians. It has the effects of clearing heat and cooling blood as well as promoting blood circulation and removing blood stasis. It mainly treats warm toxin-induced macular eruptions, heat in the blood causing vomiting and nosebleeds, amenorrhea and dysmenorrhea, and falls, blows, and pains. Additionally, it can clear and expel latent heat from the yin aspect. (Yin deficiency is manifested as five-center fever (fever in the palms, soles, and chest), dry mouth and throat, constipation, and red tongue with scanty coating, etc.).

##### The Influence of Processing on Moutan Cortex Carbon Dots

**Figure 8:**
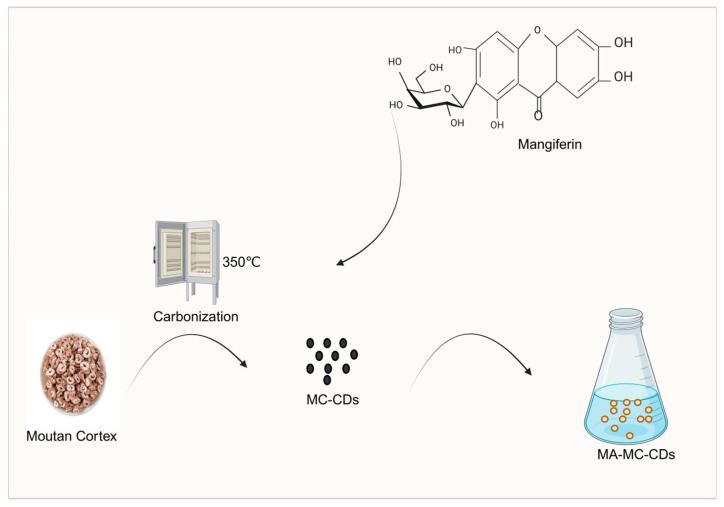
Moutan Cortex carbon dots increase MA solubility.

Mudanpi is commonly used in the treatment of blood-heat paranoia and blood stasis obstruction and is often used in conjunction with Salvia divinorum. After charcoal production, its efficacy changed from clearing and draining blood-heat to astringing and stopping bleeding. Studies have shown that during high-temperature carbonization, the original components of Moutan Cortex (e.g., danpi phenol, paeoniflorin, etc.) may be transformed into carbon dots structures with new activities. Wang Chao’er [111] analyzed the content of flavonoid components of Moutan Cortex before and after processing. The results showed that the content of flavonoids such as kaempferin, quercetin, and isorhamnetin of Moutan Cortex appeared to be significantly decreased after frying the charcoal concoction. On this basis, Jiang Guorong et al. [112] determined the content of active components in Moutan Cortex and its processed products using reversed-phase high-performance liquid chromatography (RP-HPLC), and the results showed that the contents of paeonol and paeoniflorin in carbonized Moutan Cortex were the lowest. Furthermore, Ding Anwei et al. [113] conducted further research and found that the content of paeonol in carbonized Moutan Cortex decreased with increasing processing temperature and time, and this decrease in paeonol content was highly likely to enhance the hemostatic effect of carbonized Moutan Cortex. Meanwhile, Li Xian et al. [114] pointed out that the main active components responsible for the hemostatic effect in carbonized Moutan Cortex are tannins and ethyl acetate. It can thus be seen that the hemostatic effect of carbonized Moutan Cortex is exerted by the synergy of multiple components. This conclusion indicates that the material basis of Moutan Cortex undergoes transformation during processing. In addition, carbonization of Moutan Cortex can improve the solubility and bioavailability of other components. Studies by Kong et al. [115] have shown that mangiferin (MA) has poor solubility in water; however, when carbonized Moutan Cortex is pyrolyzed to obtain Moutan Cortex carbon dots (MC-CDs), which form a saturated suspension (MA-MC-CDs) with MA, the solubility of MA in water can be improved. The main mechanism is that MC-CDs form intermolecular interactions with MA (e.g., the -OH and -NH groups on the surface of MC-CDs form hydrogen bonds with the phenolic hydroxyl groups of MA), which serves as a way to enhance the dispersibility of MA in water. At the same time, MC-CDs can regulate the pharmacokinetic process of MA in the body by promoting the gastrointestinal absorption of MA and reducing its elimination rate, thereby improving the bioavailability of MA (Figure 8).

##### Pharmacological Activity of Moutan Cortex Carbon Dots

Luo Juan et al. [116]. prepared Moutan Cortex carbonisata nano-components (MCC-NCs) from pyrolysis. After administration of MCC-NCs to a rat model of hemorrhagic fever caused by dried yeast combined with ethanol, it was found that plasma viscosity was significantly reduced, erythrocyte pressure volume, erythrocyte distribution width, hemoglobin content, and erythrocyte count were decreased, and activated partial thromboplastin time, prothrombin time, and fibrinogen level were significantly reduced. Lung tissue damage was reduced, hemorrhage, and inflammatory cell infiltration was reduced, and gastric mucosal hemorrhagic symptoms were significantly improved. This suggests that MCC-NCs can regulate bleeding-related blood status and tissue damage via activation of the endogenous coagulation pathway or the fibrinogen system, thus exerting cooling and hemostatic effects.

After processing, Moutan Cortex undergoes a transformation in its effects: on the one hand, it can enhance astringent and hemostatic effects, and on the other hand, it can improve the bioavailability of other drugs. This characteristic provides new ideas for the development of new pharmaceutical preparations. In recent years, Moutan Cortex carbon dots extracted from carbonized Moutan Cortex have attracted wide attention from researchers. Studies have found that Moutan Cortex carbon dots may exert their hemostatic effect by affecting relevant factors in the blood coagulation process and promoting platelet aggregation, thereby accelerating hemostasis.

#### 3.3.2. Scutellariae Radix (Huangqin)

Scutellariae radix is the dried root of *Scutellaria baicalensis* Georgi, family Labiatae. It is bitter in flavor and cold in nature. It belongs to lungs, gallbladder, spleen, large intestine, and small intestine meridians [71]. It enters the lungs, gallbladder, spleen, large intestine, and small intestine meridians, and possesses effects such as clearing heat and drying dampness, purging fire and removing toxicity, stopping bleeding, and preventing miscarriage [117]. Scutellariae radix was first recorded in *Shennong’s Classic of Materia Medica* and classified as a middle-grade herb; its medicinal value has been elaborated in detail in numerous ancient medical classics.

##### The Influence of Processing on Scutellariae Radix Carbon Dots

Scutellariae radix is commonly used in the treatment of lung-heat cough, damp-heat diarrhea, and carbuncle sores, and is mostly combined with Bupleuri radix in prescriptions [118]. After being processed into charcoal, its efficacy changes from clearing heat and purging fire to cooling blood and stopping bleeding. By comparing Scutellariae radix charcoal with the original Scutellariae radix, Li Yongkang et al. [119]. found that the contents of baicalin and wogonoside gradually decreased, while the contents of baicalein and wogonin increased. This indicates that during the stir-frying process of Scutellariae radix into charcoal, a glycosidic bond cleavage reaction occurs in the glycoside components, promoting the conversion of glycoside components into aglycones. The aglycones (such as baicalein) produced after processing have a simpler molecular structure and lower polarity, thus making it easier to form a uniform carbon core structure during the carbonization process. In addition, modern pharmacological studies have shown that due to changes in its chemical components, the material basis also undergoes transformation. The main component of Scutellariae radix that exerts anti-inflammatory effects is baicalin; after high-temperature processing, the content of baicalin decreases, while the content of astringent components (such as porous charcoal) increases, thereby enabling it to exert a hemostatic effect. Huang Qi [120] et al. scientifically explained the hemostatic mechanism by comparing the ellagitannin content and charcoal adsorption of Scutellariae radix before and after frying charcoal and found that its ellagitannin content decreased and charcoal adsorption increased significantly after charcoal preparation. What is more noteworthy is that water solubility occupies a key position among the many properties that enable a drug to function effectively. When the water solubility of a drug is improved, its dissolution process in the body becomes smoother, absorption efficiency is enhanced, and bioavailability of the drug is increases, allowing the drug to better perform its therapeutic efficacy. Chen Zhi et al. [27]. prepared Scutellariae radix charcoal by frying, and then successfully obtained the water-soluble carbon dots of Scutellariae radix charcoal through a series of extraction, isolation, and purification operations. It was shown that these carbon dots were able to significantly increase the solubility of baicalin and baicalein in water within the linear range. This phenomenon implies that the water solubility of baicalin was significantly improved after making it into carbon dots, which lays a good foundation for the drug to exert its therapeutic effect more efficiently.

##### Pharmacological Activity of Scutellariae Radix Carbon Dots

**Figure 9:**
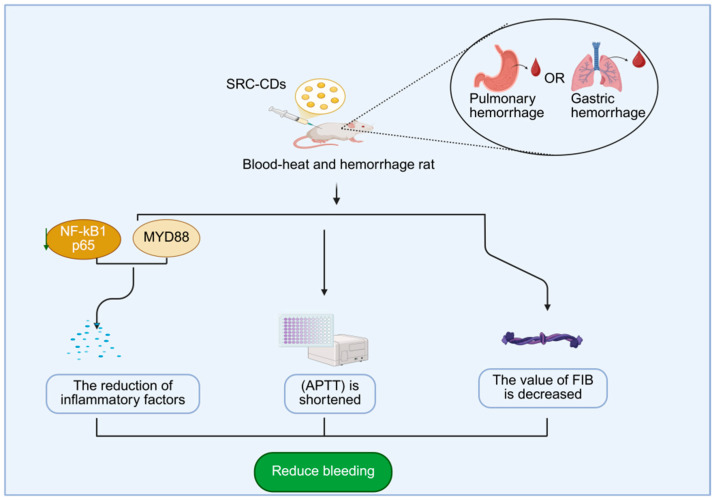
Mechanism of hemostatic action of SRC-CDs.

In recent years, it has become a research hotspot to extract carbon dots from Scutellariae radix and explore their pharmacological effects. In terms of hemostasis, Zhang [121] and colleagues extracted Scutellariae radix carbon dots (SRC-CDs) by calcination and administered SRC-CDs to a rat model of thermal hemorrhage. They found that there was a significant reduction in NF-κB p65 and myD88 protein expression, decreased inflammatory cytokine levels, shortened APTT, and decreased FIB values in the rat lung and stomach tissues. It indicates that SRC-CDs mainly improve the abnormal coagulation function by activating the endogenous coagulation pathway and fibrin system. Meanwhile, they inhibit the expression of myD88/NF-κB p65 protein signaling pathway and reduce the pathway of inflammatory factor release, thereby promoting hemostasis and blood cooling (Figure 9). In terms of anti-tumor effects, Zongjiang Luo et al. [122]. prepared baicalin carbon dots by a hydrothermal method. It was shown that during the development of canine mammary tumors, canine mammary tumor cells (e.g., CIPp and CMT-7364 cells) showed unlimited proliferation and eventually formed tumors. The CCK-8 assay showed that the proliferation of canine mammary tumor cells, such as CIPp and CMT-7364, was inhibited and the cell survival rates decreased after baicalein carbon dots were applied to the canine mammary tumor cells, suggesting that baicalein carbon dots can inhibit the proliferation of canine mammary tumor cells by affecting the proliferative process of the tumor cells.

To sum up, Scutellariae radix is not only effective in clearing heat and drying dampness in the traditional way, but the carbon dots extracted after the charcoal treatment can improve the solubility of the drug, increase the bioavailability and at the same time play pharmacological roles in hemostasis and anti-tumor, which provides a broader prospect for the development and application of traditional Chinese medicine.

### 3.4. New Drug Efficacy

#### 3.4.1. Aurantii Fructus Immaturus (Zhishi)

Aurantii Fructus Immaturus is the dried young fruit of *Citrus aurantium* L. and its cultivated varieties or *Citrus sinensis* Osbeck, the sweet orange of the Rutaceae family. It is bitter, pungent, and slightly cold. It enters the spleen, stomach, and large intestine meridians and has the effects of breaking Qi and resolving stagnation, reducing phlegm and dissipating masses, and lifting middle Qi (it mainly targets excess syndromes caused by Qi stagnation and the internal retention of food. Drugs with strong Qi-moving effects break up stagnated Qi and dissipate retained accumulations to restore the normal transportation and transformation functions of the zang-fu organs and the smooth flow of Qi.) [71]. Modern research shows that Aurantii Fructus Immaturus can promote gastrointestinal peristalsis, relieve smooth muscle spasm, and has certain cardiotonic and antihypertensive effects, commonly used in functional dyspepsia and gastrointestinal dyskinesia.

##### The Influence of Processing on Aurantii Fructus Immaturus Carbon Dots

Aurantii Fructus Immaturus, as a traditional Chinese medicine commonly used in the treatment of gastrointestinal diseases and chest paralysis, is mostly used to break down Qi and eliminate accumulation, and to dissolve phlegm and dissipate lumps. During the process of making charcoal, the chemical composition and properties of the Aurantii Fructus Immaturus are altered, weakening its original functions of breaking Qi, eliminating accumulation, resolving phlegm, and relieving fullness, while highlighting its astringent and hemostatic effects. In the study of processed products of Aurantii Fructus Immaturus, Wang Wenka’s team [123] used the HPLC method to determine the content of naringin and neohesperidin in different processed products. The results showed that the content of these two components were the lowest after stir-frying into charcoal. Similarly, Ouyang Rong [124] investigated the content of synephrine in different processed products and found that the content of synephrine decreased in charcoal-processed Aurantii Fructus Immaturus (Table 8). From the perspective of modern pharmacological research, hesperidin (a flavonoid component) can reduce capillary permeability and exert an anti-inflammatory effect, while synephrine and N-methyltyramine are key components for blood pressure elevation and anti-shock. However, after Aurantii Fructus Immaturus is processed into charcoal by stir-frying, the contents of the above-mentioned components all decrease significantly, which may affect the pharmacological activities of its carbon dots in anti-inflammation, anti-shock, and other aspects. In addition, Aurantii Fructus Immaturus undergoes high temperature during the process of charcoal processing, which may generate nano-sized components, leading to the transformation of the pharmacodynamic material basis of Aurantii Fructus Immaturus. Wang Meijun’s team [125] found that the analgesic and antidepressant effects of Aurantii Fructus Immaturus were enhanced after charcoal processing, which may be related to the newly generated nano-components.

##### Pharmacological Activity of Aurantii Fructus Immaturus Carbon Dots

**Figure 10:**
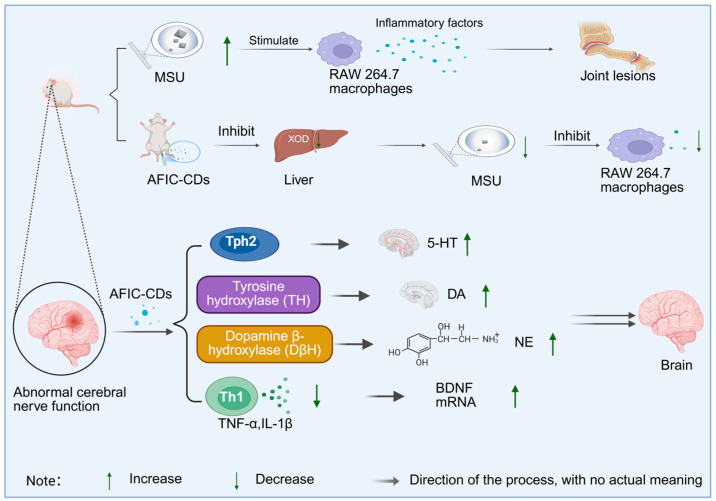
Mechanism of anti-gout and antidepressant action of AFIC-CDs.

Extracting carbon dots from charred Aurantii Fructus Immaturus and studying their pharmacological effects is expected to open up new directions for clinical application. Compared with raw Aurantii Fructus Immaturus after carbonization and conversion into carbon dots, charred Aurantii Fructus Immaturus not only retains some of its original active ingredients but also exhibits new pharmacological functions. Wang et al. [126] prepared Aurantii Fructus Immaturus carbon dots (AFIC-CDs) using a one-step pyrolysis method and studied their role in anti-hyperuricemia and gout. Patients with hyperuricemia often present with elevated serum uric acid levels accompanied by joint (such as ankle) lesions. Monosodium urate crystals (MSU) can stimulate RAW 264.7 macrophages and promote the production of inflammatory factors IL-1β and TNF-α. After intervention with AFIC-CDs, the levels of serum uric acid, IL-1β, and TNF-α in the model group decreased significantly, indicating that AFIC-CDs can reduce uric acid production by inhibiting the activity of xanthine oxidase (XOD) in the liver and serum and suppress the release of inflammatory factors, thereby exerting a therapeutic effect. Compared with the extract of raw Aurantii Fructus Immaturus, AFIC-CDs show stronger XOD inhibitory activity and anti-inflammatory effect with a lower effective dose. Li et al. [127] also prepared AFIC-CDs via pyrolysis and explored their potential in anti-depression. Patients with depression often exhibit symptoms such as low mood, decreased interest, fatigue, and inattention, accompanied by abnormal brain neural functions, including disorders in the levels of neurotransmitters serotonin (5-HT), dopamine (DA), and norepinephrine (NE), as well as abnormal levels of inflammatory factors IL-1β and TNF-α. The expression of brain-derived neurotrophic factor (BDNF) and tryptophan hydroxylase 2 (Tph2) in the cerebral cortex also tends to change [128]. After treatment with AFIC-CDs, the levels of 5-HT, DA, and NE in the cerebral cortex of mice increased, the levels of IL-1β and TNF-α decreased, and the mRNA expressions of BDNF and Tph2 were also significantly upregulated. The improvement of the above indicators indicates that AFIC-CDs can alleviate depression-related neural dysfunction by regulating neurotransmitter levels, inhibiting neuro-inflammation, and promoting the expression of factors related to neuroplasticity, as shown in Figure 10. Compared with raw Aurantii Fructus Immaturus, AFIC-CDs can more effectively cross the blood–brain barrier, act directly on the central nervous system, and show unique advantages in multi-transmitter and multi-pathway regulation.

In summary, Aurantii Fructus Immaturus can effectively regulate the Qi of the gastrointestinal tract and alleviate the symptoms of bloating and fullness in the stomach and abdomen, as well as the accumulation of undigested food. With deepening research on Aurantii Fructus Immaturus, researchers have successfully extracted carbon dots from the charcoal of Aurantii Fructus Immaturus, a discovery that further expands the medicinal potential of AFIC-CDs. It has been confirmed that the extracted AFIC-CDs undergo significant changes in its chemical composition. These changes have conferred a series of new pharmacological effects on AFIC-CDs, demonstrating unique advantages in the treatment of several diseases. In neurological diseases, AFIC-CDs have shown antidepressant effects; in gout-related diseases, AFIC-CDs have demonstrated significant anti-hypouricemic and anti-gout activity. In addition, AFIC-CDs have shown some potential in anti-inflammation.

#### 3.4.2. Ginger (Shengjiang)

In the field of traditional Chinese medicine, ginger is a widely used and time-honored medicinal herb derived from the fresh rhizome of *Zingiber officinale* Rosc. It is slightly warm in nature, pungent in flavor, and belongs to the lung, spleen, and stomach meridians, and has a wide range of efficacies such as relieving symptoms and dispersing cold, warming the body, and stopping vomiting [71]. Ginger is classified as an intermediate product in *Mingyi Bielu* (Supplementary Records of Famous Physicians), which describes the origin, form, and some of the medicinal effects of ginger. Since then, many pharmacological texts, such as the *Compendium of Materia Medica*, have elaborated on ginger, enriching people’s knowledge of its medicinal value.

##### The Influence of Processing on Ginger Carbon Dots

Ginger is commonly used in the treatment of cold and flu, cold spleen and stomach, cold stomach and vomiting, cold phlegm cough, and other diseases. The medicinal property of ginger is pungent in taste and warm in nature, and it “moves freely without stagnation”. After being processed into ginger charcoal, its pungent taste disappears, and its nature becomes retaining rather than moving. Its efficacy changes from the original effects—such as relieving exterior symptoms to dispel cold and warming the middle energizer to stop vomiting—to focusing primarily on astringing to stop bleeding. Studies have shown that during the process of ginger being processed into ginger charcoal, high temperatures cause partial chemical components to decompose and transform, leading to a significant reduction in the content of volatile oil. Sun Meng et al. [129]. found that new components (dibutyl phthalate, catechol, gingerone, and 1,2,4,5-tetrahydroxyphenol) were generated during the process of ginger charcoal through a preliminary analysis of the chemical composition of ginger charcoal. Li Juan [130] extracted the volatile components of fresh ginger and ginger charcoal using the steam distillation method and found that the content of low-boiling-point volatile components in ginger charcoal decreased significantly. These changes in components lead to a transformation in the material basis of ginger after processing. Li Tianye’s team [131] evaluated the gingerols in fresh ginger and ginger charcoal and found that the contents of gingerols (zingerone, 6-gingerol, and 8-gingerol) in ginger charcoal were significantly higher than those in fresh ginger. Since gingerols are the main components responsible for antioxidant activity, this enhances the antioxidant activity of ginger charcoal, thereby providing a material basis for in-depth research on the application of ginger carbon dots in antioxidant-related fields (Table 9).

##### The Pharmacological Activity of Ginger Carbon Dots

**Figure 11:**
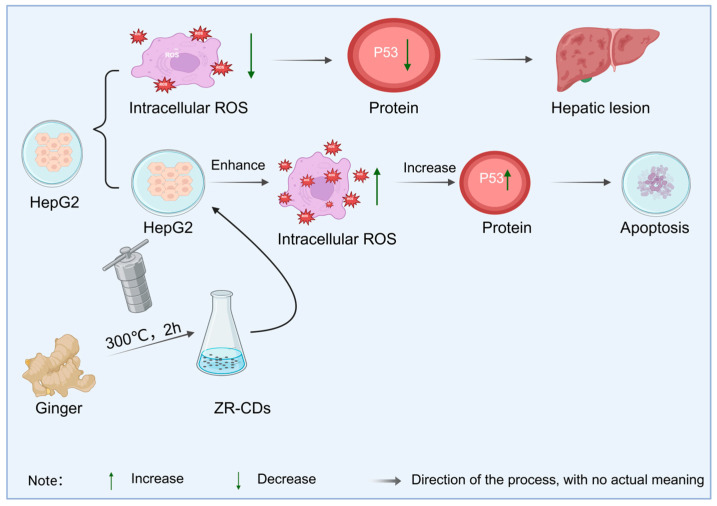
Inhibition of HepG2 cell proliferation by ginger carbon dots.

As a typical natural medicine, ginger-derived carbon dots not only provide strong evidence for the connection between natural medicines and carbon-structured compounds, but also highlight significant advantages over traditional ginger. Studies have shown that carbon dots synthesized from ginger as a precursor via green methods such as one-step pyrolysis or the hydrothermal method, while inheriting the natural active ingredients of ginger, generate new pharmacological activities by virtue of the synergistic effect between the carbon dot structure and surface functional groups. Zhang et al. [132] found that the ginger carbon dots (ZR-CDs) obtained in this way can effectively inhibit pain responses caused by chemical and thermal stimuli by regulating the opioid system and serotonin levels, thus proving that ginger carbon dots can produce analgesic effects. In addition, the work of Li et al. [133] further revealed the unique value of ginger carbon dots in anti-tumor mechanisms. The ginger carbon dots they prepared can not only inhibit the proliferation of hepatocellular carcinoma cells and promote apoptosis by increasing intracellular ROS levels and upregulating the expression of p53 protein (as shown in Figure 11), but mass spectrometry analysis also identified that natural active molecular structures such as curcumin were still retained in the carbon dots. This finding is of great significance, indicating that natural medicine carbon dots are not simple carbon carriers but result from the synergistic effect between carbon nano-skeletons and active ingredients of natural medicines.

Ginger is often used in the treatment of various diseases, exerting important effects such as relieving exterior syndrome and dispelling cold, warming the middle energizer to stop vomiting, etc. After high-temperature processing, ginger is transformed into ginger charcoal, with significant changes in its properties and efficacy. Ginger charcoal not only inherits some characteristics of ginger but also has good antioxidant properties. More importantly, carbon dots can be extracted from ginger charcoal, and these carbon dots can produce good analgesic and anti-cancer effects. Therefore, whether ginger carbon dots can be used as a new type of alternative analgesic drug deserves further research and attention.

#### 3.4.3. Artemisiae Argyi Folium (Aiye)

Artemisiae argyi Folium is the dried leaves of *Artemisia argyi* Levl. et Vant [71] of the Asteraceae family. It is bitter, pungent, and warm in nature, and belongs to the liver, spleen, and kidney meridians, with the effect of warming menstruation, stopping bleeding, dispersing cold, and relieving pain.

##### The Influence of Processing on Artemisia Argyi Folium Carbon Dots

As a commonly used Chinese medicine, Artemisia argyi Folium shows unique value in the treatment of gynecological diseases, rheumatism and paralysis, and skin diseases. Jiang Liping [134] examined the eucalyptus oleoresin content of Artemisia argyi Folium and charcoal of Artemisia argyi Folium and found that the volatile component (eucalyptus oleoresin) content of Artemisia argyi Folium disappeared after frying to charcoal. Gule [135] explored the chemical composition of the charcoal of Artemisia argyi Folium and isolated two flavonoids, namely quercetin and eicosanol (Table 10). Modern pharmacological studies have shown that the active components (such as volatile oils and flavonoids) contained in Artemisia argyi Folium can not only regulate uterine contractions and improve blood circulation but also exert antibacterial and anti-viral effects. After high-temperature carbonization, although the volatile components disappear, some core components (e.g., flavonoids) are retained; therefore, carbonized Artemisia argyi Folium also possess antibacterial and anti-inflammatory effects. In addition, by comparing the microscopic characteristics of Artemisia argyi leaves and carbonized Artemisia argyi leaves, Li Xining [136] found that a large amount of carbon is generated after Artemisia argyi Folium is stir-fried into charcoal; at the same time, a significant reduction in insoluble calcium oxalate cluster crystals was also observed. The reason for this lies in the production of free calcium ions (Ca^2+^) during the high-temperature carbonization process, and Ca^2+^ can promote blood coagulation. Thus, the hemostatic function of Artemisia argyi Folium is enhanced after stir-frying into charcoal, providing an ideal precursor for the preparation of carbon dots.

##### Pharmacological Activity of Artemisia Argyi Folium Carbon Dots

**Figure 12:**
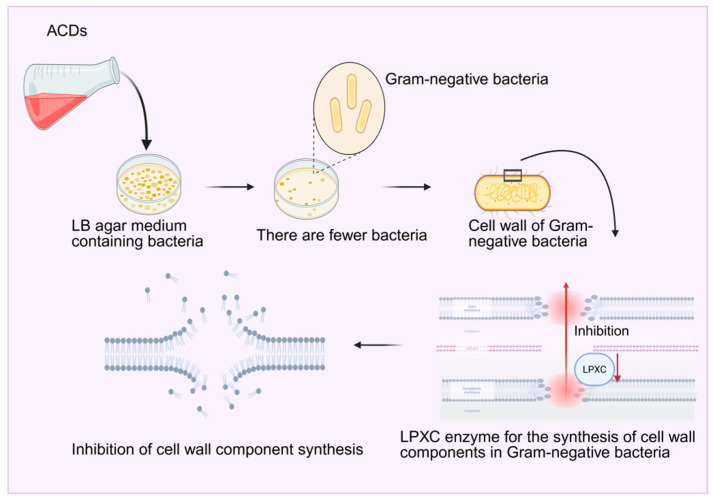
Diagram of the antimicrobial mechanism of ACDs.

Studies have found that carbon dots derived from charred Artemisia argyi Folium not only completely retain the antibacterial and antioxidant properties of Artemisia argyi Folium but also exhibit novel pharmacological potential. In terms of antibacterial activity, Wang et al. [137] prepared Artemisia argyi Folium carbon dots (ACDs) by simulating smoking: the smoke from dried and ignited Artemisia argyi Folium was introduced into deionized water, and reddish-brown ACDs were obtained after filtration and dialysis. The growth of Gram-negative bacteria was inhibited on LB agar plates and liquid media containing ACDs, as evidenced by a reduction in the number of colonies and a decrease in the optical density value (OD_600_). Further studies revealed that ACDs can damage the cell wall of Escherichia coli, inhibit biofilm formation, reduce the activity of LPXC (a key enzyme in cell wall synthesis), and alter its secondary structure. These results indicate that ACDs inhibit Gram-negative bacteria through multiple pathways, including disruption of the bacterial growth environment, affecting physiological processes, selective damage to cell walls, interference with biofilm formation, and inhibition of the synthesis of cell wall components. Li Qiongyang et al. [138] also studied the antibacterial activity of Artemisia argyi Folium carbon dots (AAFC-CDs) and found that they exhibit inhibitory effects on Gram-positive bacteria (such as Staphylococcus aureus). Adding AAFC-CDs to bacterial suspensions can cause shrinkage and deformation of the bacterial cell wall, increase membrane permeability, and the minimum inhibitory concentration (MIC) against Staphylococcus aureus was determined to be 1.25 mg/mL. The results showed that AAFC-CDs inhibited bacterial growth by damaging the cell wall structure and altering membrane permeability, maintaining bacteria in a state of apoptosis for an extended period. In addition, studies have confirmed through a series of experiments that the antioxidant capacity of AAFC-CDs is stronger than that of raw Artemisia argyi Folium, as shown in Figure 12. In addition to antibacterial and antioxidant activities, AAFC-CDs also exhibited anti-frostbite pharmacological effects. Kong et al. [139] found in a mouse frostbite model induced by an ice-water bath that frostbite can lead to damage to cell structure and function, decreased enzyme activity, slowed energy metabolism, and trigger platelet aggregation, vascular thrombosis, and increased levels of inflammatory mediators during rewarming; repeated freezing and thawing further exacerbate tissue damage. After treatment with AAFC-CDs, the degree of tissue damage and necrosis in the ears of mice was significantly reduced, the levels of inflammatory factors IL-1β and TNF-α decreased, the degree of limb stiffness (reflected by changes in grip strength) improved, and the increase in blood glucose caused by frostbite was also significantly reduced. These results indicate that AAFC-CDs exert anti-frostbite effects by alleviating tissue damage, inhibiting inflammatory responses, and mitigating frostbite-induced hyperglycemia.

In summary, based on retaining the original antibacterial and antioxidant functions of Artemisia argyi Folium, AAFC-CDs have further expanded new pharmacological activities, such as anti-frostbite.

## 4. Conclusions

Before TCMs are applied in clinical practice, they often undergo processing to enhance or alter their medicinal effects. Among numerous processing theories, “stir-frying into charcoal while retaining the inherent property” is an important one. Through high-temperature carbonization, the surface of the medicinal materials becomes charred black while the interior retains its inherent properties, aiming to achieve specific effects such as hemostasis. However, the traditional theory “Blood ceases bleeding upon encountering blackness (blackness refers to carbonized Chinese herbs, and this theory essentially means that carbonized herbs exert a hemostatic, bleeding-stopping effect)” cannot fully explain the diverse pharmacological mechanisms of various charred medicines. Studies have found that new substances, which do not exist in the original medicinal materials, emerge during the processing of charred medicines. This indicates that chemical composition transformation and new substance generation may occur during the charring process, providing a new perspective for interpreting the scientific connotation of “burn as charcoal with function preserved”. It is noteworthy that the high-temperature carbonization process used for preparing charred medicines is highly consistent with the synthesis method of TCM-CDs. CDs prepared using Chinese medicinal materials as precursors not only inherit the advantages of carbon dot materials themselves, such as small size and high biocompatibility, but also significantly enhance the pharmacological activity of the original medicinal materials, achieve multi-target synergistic therapy, improve the bioavailability of drugs, and even endow new medicinal functions.

Although TCM-CDs show broad prospects for clinical application, their transition from laboratory research to large-scale industrial production and clinical translation still face many challenges. Due to the fact that Chinese medicinal materials themselves are affected by factors such as producing area, harvesting season, and processing methods, there are fluctuations in their chemical components. This makes it difficult to standardize the quality of raw materials used as precursors for carbon dots, which in turn affects the consistency of the size, surface functional groups, and biological activity of TCM-CDs. To solve this problem, it is necessary to establish a strict raw material quality control system or use standardized extracts as precursors for carbon dot synthesis. Only by systematically solving these problems and comprehensively evaluating their biological safety can TCM-CDs be produced on a large scale.

## Figures and Tables

**Table 1:** Top-down approach.

Method	Operation Mode	Result
Arc discharge	The carbon electrode discharges in an inert gas and the material evaporates and condenses.	Higher purity but more impurities
Electrochemical	The electrodes dissolve and aggregate when an electric current is passed through the electrolyte.	Metal and oxide nanomaterials with easily controllable morphology
Laser ablation	The laser irradiates the carbon target to vaporize and condense it.	Small and well-dispersed, but associated with high equipment cost

**Table 2:** Bottom-up approach.

Method	Operation Mode	Result
Hydrothermal	In high-temperature and high-pressure aqueous solutions, reactants undergo dissolution, reaction, and crystallization to form the required materials.	Uniform particle size and high crystallinity
High-temperature pyrolysis	Under high-temperature and inert gas conditions, the precursor undergoes decomposition reactions to form the required materials.	Higher purity
Solvothermal	Organic solvents are used instead of water to carry out high-temperature and high-pressure reactions in a closed reactor, promoting the synthesis of substances.	Dissolve some insoluble reactants in the hydrothermal method
Microwave-assisted carbonization	Rapid heating of reactants using microwave radiation to induce carbonization reactions.	Fast reaction speed, uniform product structure, and relatively low energy consumption
Template method	Guide the growth of materials with the help of templates to form specific structures.	Precise control of morphology, size, and porosity
Acid dehydration	Dehydration and condensation of reactants to form target products.	Commonly used in the synthesis of organic materials

**Table 3:** Carbon dots hemostatic pathways of Phellodendri Cortex.

Avenues	Relevant Indicators	Brief Description of Mechanism of Action
Coagulation pathway	Associated with TT and FIB levels	Increase FIB levels and shorten TT
Blood platelet	-----	Upgrade the number of PLTs

**Table 4:** Changes in chemical composition of rhubarb after concocting.

Ingredient	Rhubarb Charcoal
5-Hydroxymethylfurfural	Additional
Total flavonoids	Minimize
Total anthraquinone	Minimize
Rhubarb phenol, rhubarbol, rhubarbic acid, and rhubarbin methyl ether	Minimize

**Table 5:** Mechanisms of action of the hemostatic pathway.

Avenues	Relevant Indicators	Brief Description of Mechanism of Action
Endogenous coagulation	APTT	Reduce APTT values
Exogenous coagulation	PT	Lower PT
Coagulation	TT and FIB	Increase FIB levels and shorten TT
PLT	-----	Boost platelet count

**Table 6:** Changes in chemical composition of Salvia miltiorrhiza after concocting.

Ingredient	Salvia Miltiorrhiza Charcoal	Impact on Efficacy
Salvianolic acid B	Significantly lower	Affects antioxidant, anticoagulant, etc.
Tanshinone IIA		Impact on antimicrobial and anti-inflammatory properties

**Table 7:** Comparison of licorice and Gly-CDs in anti-viral properties.

Program	Licorice	Gly-CDs
Focus on role	Regulation of viral replication cycle, body inflammation, immunity, and oxidative stress	Multi-point virus suppression
Anti-RSV site of activity and stage of action	n-butanol and aqueous sites, acting in the adsorption, penetration, and replication phases	/
Anti-viral or anti-RSV specific modalities	Inhibition of related pathways attenuates inflammation; Increased IFN-β inhibits immune escape; Reduced oxidative stress	Binding viruses to inactivate, inhibit invasion, disrupt replication, activate immunity, and inhibit ROS and regulatory factors
Anti-viral spectrum	Inhibits five viruses	Effective against a wide range of viruses
Role advantages	/	Multi-target synergy, large surface area, multiple sites, and multivalent action

**Table 8:** Changes in the content of charcoal components in Aurantii Fructus Immaturus.

Ingredient	Zhishi Charcoal	Impact on Efficacy
Naringin	Lower	May weaken the effect of reducing capillary permeability and anti-inflammatory
Neohesperidin		
Simferin	Substantial reduction	May diminish pressor-boosting and anti-shock effects

**Table 9:** Comparison of chemical composition-related indexes between ginger and ginger charcoal.

Ingredient	Fresh Ginger	Ginger Charcoal	Evolution
Phenol	None	Catechol and 1,2,4,5-tetrahydroxyphenol	Additional
Salts	None	Dibutyl phthalate	Additional
Volatility	Contains a variety of low-boiling-point volatile ingredients	Significant reduction in low-boiling point volatile components	Minimize
Curcumin	Gingerone not detected; 6-gingerol, 8-gingerol, etc., in low content	Gingerone, 6-gingerol, and 8-gingerol were significantly higher than ginger	Gingerone from scratch and other ingredients increased

**Table 10:** The chemical composition changes in Artemisia argyi Folium after processing.

Ingredient	Charred Artemisia Argyi Folium	Impact on Efficacy
Flavonoids	Addition of quercetin and eicosanoids	/
Volatile oil (in general)	Disappearance of eucalyptus oil extract	Affects the regulation of uterine contractions, improves blood circulation, and may also affect antimicrobial capacity
Carbon	Additional	May give new properties such as hemostasis to mugwort charcoal
Refractory calcium oxalate clusters	Substantial reduction	Produces Ca^2+^, which can promote blood clotting
